# Effect of Placenta-Derived Mesenchymal Stromal Cells Conditioned Media on an LPS-Induced Mouse Model of Preeclampsia

**DOI:** 10.3390/ijms23031674

**Published:** 2022-01-31

**Authors:** Anna Maria Nuzzo, Laura Moretti, Paolo Mele, Tullia Todros, Carola Eva, Alessandro Rolfo

**Affiliations:** 1 Department of Surgical Sciences, University of Turin, 10126 Turin, Italy; a.nuzzo@unito.it (A.M.N.); l.moretti@unito.it (L.M.); tullia.todros@unito.it (T.T.); 2 Department of Neurosciences, Neurosciences Institute Cavalieri Ottolenghi (NICO), San Luigi Hospital, University of Turin, Orbassano, 10043 Turin, Italy; paolo.mele@unito.it (P.M.); carola.eva@unito.it (C.E.)

**Keywords:** placenta-derived mesenchymal stromal cells, preeclampsia, mouse model, placenta

## Abstract

We tested the pro-angiogenic and anti-inflammatory effects of human placenta-derived mesenchymal stromal cells (hPDMSCs)-derived conditioned media (CM) on a mouse model of preeclampsia (PE), a severe human pregnancy-related syndrome characterized by maternal hypertension, proteinuria, endothelial damage, inflammation, often associated with fetal growth restriction (FGR). At d11 of pregnancy, PE was induced in pregnant C57BL/6N mice by bacterial lipopolysaccharide (LPS) intravenous injection. At d12, 300 μL of unconditioned media (control group) or 300 μL PDMSCs-CM (CM group) were injected. Maternal systolic blood pressure was measured from 9 to 18 days of pregnancy. Urine protein content were analyzed at days 12, 13, and 17 of pregnancy. At d19, mice were sacrificed. Number of fetuses, FGR, fetal reabsorption, and placental weight were evaluated. Placentae were analyzed for sFlt-1, IL-6, and TNF-α gene and protein expressions. No FGR and/or reabsorbed fetuses were delivered by PDMSCs-CM-treated PE mice, while five FGR fetuses were found in the control group accompanied by a lower placental weight. PDMSCs-CM injection significantly decreased maternal systolic blood pressure, proteinuria, sFlt-1, IL-6, and TNF-α levels in PE mice. Our data indicate that hPDMSCs-CM can reverse PE-like features during pregnancy, suggesting a therapeutic role for hPDMSCs for the treatment of preeclampsia.

## 1. Introduction

The preeclamptic syndrome (PE), exclusive to human pregnancy, represents the main cause of fetal–maternal mortality and morbidity worldwide [1,2]. PE generally resolves at delivery with placenta removal, but it causes severe long-term complications for both the mother and the fetus, such as cardiovascular and neurological disorders, diabetes, and metabolic syndrome [3]. Despite almost three decades of intensive investigation, PE still remains an unsolved medical need.

Indeed, preeclampsia has a major social–economic impact due to the lack of effective therapies, except for a timely and often premature delivery. There were several unsuccessful attempts to find a resolutive cure for PE, ranging from new drug candidates to drug relocation. The main problem with preeclampsia is that, as a syndrome, it is multifactorial, a destructive mix of inflammation, endothelial damage, and immunological impairment [2,4]. Key features of PE are a maternal immune maladaptation towards the fetoplacental district with a shift towards Th1 immunity [5,6,7], increased placental release of proinflammatory cytokines (e.g., Tumor Necrosis Factor-α—TNF-α; Interleukin-6–IL-6), and anti-angiogenic factors (e.g., soluble FMS-like tyrosine kinase-1—sFlt-1) that promote aberrant placental angiogenesis and generalized endothelial cell activation and damage [8,9,10,11]. Therefore, an ideal PE therapeutic approach must be able to contemporarily target all preeclamptic culprits and not to just mitigate a single clinical symptom as hypertension or inflammation.

The human placenta has been identified as a source of mesenchymal stromal cells (placenta-derived mesenchymal stromal cells—PDMSCs). PDMSCs could be isolated from the chorionic villi, the amnion, and the decidua and possess an increased self-renewal potential. Moreover, PDMSCs express stem cell markers (e.g., OCT-4, NANOG) and could differentiate into condrogenic, adipogenic, and osteogenic lineages [12,13,14]. Importantly, PDMSCs are characterized by unique immunologic and immune-regulatory properties, thus exerting a powerful immunosuppressive effect on T-cells [15,16,17,18]. Placental MSCs have been shown to promote angiogenic growth and to possess anti-inflammatory, anti-fibrotic, and cytoprotective abilities mediated by both direct cell-to-cell contact and/or specific trophic mediators more than cell differentiation [15,19].

Thus, PDMSCs may be an attractive therapeutic candidate for PE treatment. Recently, decidual MSCs were injected in vivo in a Th1 cell-induced PE-like mouse model demonstrating the ability to ameliorate PE-like symptoms as blood pressure and proteinuria [20]. In line with these results, an endotoxin-induced PE rat model infused with umbilical cord blood-derived MSCs showed decreased blood pressure, proteinuria, and inflammation relative to untreated controls [21]. Finally, commercially available placental mesenchymal cells were administered to hypertensive TLRs-induced pregnant mice, decreasing blood pressure, placental injury, and inflammation [22]. MSC-based therapy definitely sounds an intriguing potential multitarget therapeutic tool for preeclampsia.

Nevertheless, it could be hazardous to hypothesize a cell therapy for such a sensitive and delicate environment as human pregnancy for both ethical and biosafety reasons. No long-term studies on MSCs oncogenic potentials are available, and data about MSCs ability to invade maternal organs are contrasting [21,23,24,25,26,27].

Since mesenchymal stromal cells exerts their beneficial effects mainly through the release of trophic mediators, in the present study we tested the hypothesis that PDMSCs’ conditioned media (CM) could be used as an effective, ethical, and safe therapeutic approach for preeclampsia. Therefore, we evaluated the effects of PDMSCs-CM administration on maternal blood pressure, proteinuria, fetal outcome, and placental expression of sFlt-1, TNF-α, and IL-6 in an LPS-induced mouse model of preeclampsia.

## 2. Results

### 2.1. PDMSCs Presented Proper Mesenchymal Stromal Cell Profile

PDMSCs used for CM preparation presented proper mesenchymal stromal phenotype as assessed by flow cytometry. As previously published, cells were positive for CD105, CD166, CD90, and CD73, and negative for HLA-II, CD34 and CD45 (hematopoietic markers), and CD133 and CD31 (endothelial progenitor markers). PDMSCs were also negative for B cells, neutrophils, and macrophages markers CD20 and CD14 and for trophoblast and epithelial marker CD326, thus excluding any type of contamination [19,28]. RT-PCR detected the expression of typical stemness markers Oct-4 and Nanog in all PDMSCs cell lines [19,28].

### 2.2. Characteristics of the Study Population

Female pregnant mice from both control and CM group did not display significant differences in body weight after LPS injection and treatment with unconditioned/conditioned media (CM = median 27.4 g; controls: median 26.3 g). Mice from the control group showed adverse pregnancy outcomes, including fetal absorption (n = 5) and significant lower placental weight (*p* < 0.01), compared with PDMSCs-CM group (Table 1). Moreover, one case of miscarriage was observed in the control group. No significant differences were observed in fetal weight (Table 1).

RBC (red blood cells) and WBC (white blood cells) count, Htc (hematocrit), and hemoglobin concentration did not change between groups but there was a trend of decrease in Plt (platelets) count in control relative to CM-treated mice (Table 1).

### 2.3. PDMSCs Conditioned Media Ameliorated Maternal Hypertension and Proteinuria in LPS-Induced PE Mouse Model

**Figure 1:**
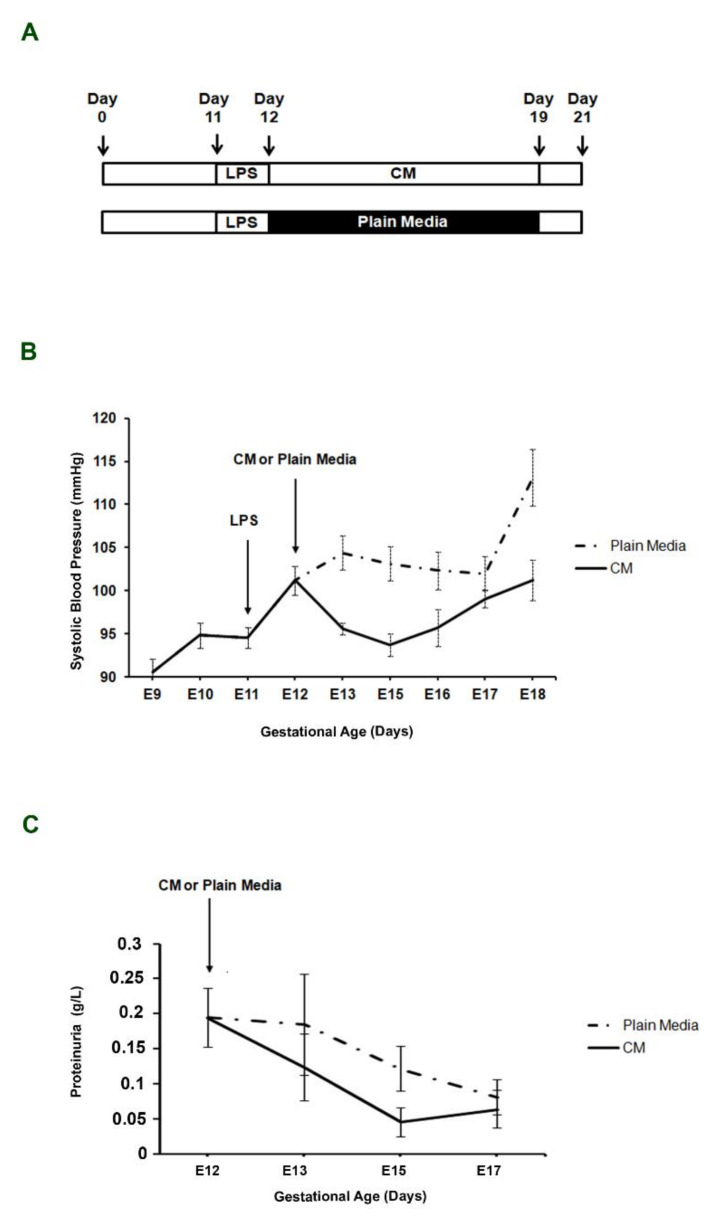
Effects of PDMSCs-CM treatment on maternal parameters during days 11 to 18 of gestation. (**A**) Study design. Blood pressure (**B**) and proteinuria (**C**) in LPS-induced PE mouse model injected with PDMSCs-CM or plain media.

We first investigated whether LPS injection was able to induce maternal hypertension. Average maternal basal SBP at days 9–11 was 93.3 ± 1.3 mmHg and it significantly increased to 101.3 ± 1.4 mmHg at day 12 (*p* = 0.04), 24 h after LPS injection (Figure 1B). We next examined the effect of plain media or PDMSCs-CM injection in LPS-induced hypertensive pregnant mice. In control mice (LPS + plain media), maternal SBP continued to increase at day 13 (104.4 ± 1.9) and day 18 (113 ± 2.3), while in CM pregnant females (LPS + PDMSCs-CM), SBP significantly decreased at days 13 (95.6 ± 0.63 mmHg, *p* < 0.01), 15 (95.7 ± 1.2 mmHg, *p* < 0.01), 16 (95.7 ± 2.1 mmHg, *p* = 0.03), and 18 (101.2 ± 2.3 mmHg, *p* < 0.01) relative to control mice (Figure 1B).

On day 12, after LPS injection and before CM or plain media injection, mean mice urine protein concentration was 0.19 ± 0.04 μg/μL. In LPS pregnant females treated by plain media, proteinuria showed a trend of decrease from day 13 to 17 relative to day 12, even though it was not significant and less dramatic compared to CM-treated mice (Figure 1C). Proteinuria decreased on d13 (0.12 ± 0.04, *p* > 0.05), d15 (0.04 ± 0.02, *p* = 0.016), and d17 (0.06 ± 0.02, *p* = 0.045) relative to day 12 CM mice (Figure 1C).

Finally, in order to investigate the effects of plain media or PDMSCs-CM infusion on liver and renal functions in LPS-induced hypertensive pregnant mice, we tested serum levels of selected parameters. On day 19, no differences were found in CM group relative to control in AST (aspartate aminotransferase) (39.75 ± 7.31 mg/dL versus 39.75 ± 5.17, *p* > 0.05) and ALT (alanine transaminase) (247.75 ± 62.88 mg/dL versus 263.75 ± 34.47 mg/dL, *p* > 0.05) levels, used as markers of liver functionality, nor in creatinine (0.0175 ± 0.01 mg/dL versus 0.07 ± 0.01 mg/dL, *p* > 0.05) and urea levels (35.25 ± 5.72 versus 34.25 ± 2.28 mg/dL, *p* > 0.05), used as markers of kidney function.

### 2.4. Placental sFlt-1, TNF-α, and IL-6 Expression Were Inhibited by PDMSCs-CM in PE Mice

**Figure 2:**
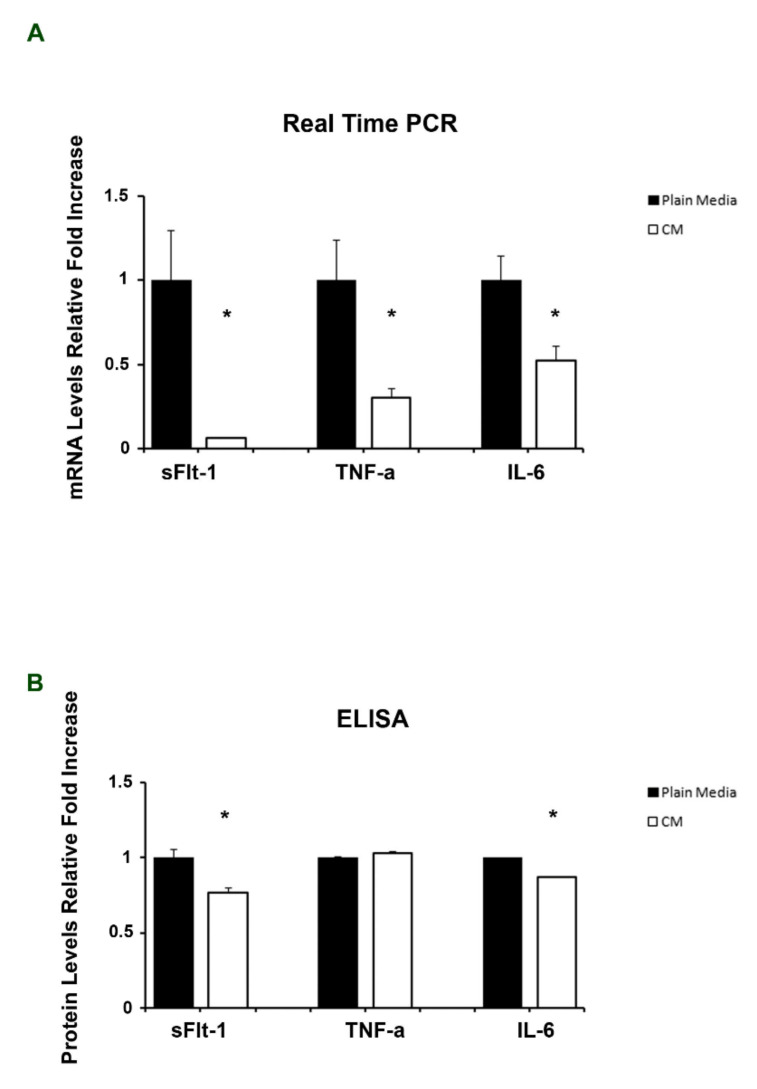
Effects of PDMSCs-CM treatment on placental sFlt-1, TNF-α, and IL-6 expression. Gene (**A**) and protein (**B**) sFlt-1, TNF-α, and IL-6 expression in placentae from LPS-induced PE mouse model injected with PDMSCs-CM or plain media. Statistical significance (*) has been considered as *p* < 0.05.

In order to determine if PDMSCs-CM treatment was effective also at the placental level, we evaluated placental expression of sFlt-1, TNF-α, and IL-6, key hallmarks of preeclampsia, in CM and control mice. We found a significant reduction of mRNA expression for the three molecular targets investigated, namely sFlt-1 (5.5-fold decrease, *p* = 0.04), TNF-α (6.2-fold decrease, *p* = 0.03), and IL-6 (5-fold decrease, *p* = 0.03) in CM mice compared to controls (Figure 2A). Decreased sFlt-1 (*p* = 0.013, 1.29-fold decrease) and IL-6 (*p* = 0.034, 1.14-fold decrease) expression in the placentae of CM mice, compared to controls, was confirmed also at the protein level (Figure 2B). No significant differences in TNF-α protein level were found in CM compared to control placentae (Figure 2B). 

## 3. Discussion

Although the etiology of preeclampsia is still elusive, recent evidence has demonstrated that MSCs of different origins, when directly infused in animal models, are able to ameliorate PE-like symptoms, thus suggesting their potential role as therapeutic agents [20,21,24]. Nevertheless, systemic administration of living cells implies significant biosafety and ethical issues that must be considered when designing therapies for such a sensitive environment as pregnancy. Intravenously injected GFP-labeled umbilical cord MSCs were detected in the renal parenchyma and placenta of PE pregnant rats and in fetal kidneys, liver, lungs, and heart [21]. Moreover, locally administered cells often die before they significantly contribute to the healing response due to poor diffusion of nutrients and oxygen [29]. Alternative approaches are therefore mandatory.

The conditioned media obtained from MSCs consist of biologically active molecules whose function is to simultaneously modulate key biological mechanisms such as inflammation and immune response, angiogenesis, cell proliferation, apoptosis, and senescence [19,30,31,32,33]. Therefore, MSCs-derived CM can be investigated as an alternative approach to cell therapy.

MSCs have been successfully isolated from a variety of tissues, including adipose tissue, pancreas, and umbilical cord blood [34,35,36]. In the present study, we used physiological MSCs derived from placental chorionic villi because of their immunosuppressive, pro-angiogenic, anti-inflammatory, and cytoprotective activities potentially useful against the aberrant placentation typical of PE [19,31,35,37,38,39]. In our previous works we found that PDMSCs-CM is able to modulate in vitro the expression of inflammatory cytokines, angiogenic factors, senescence markers, and cell cycle modulators in the placental villi [19,31].

Herein, we performed intravenous PDMSCs-CM administration to investigate the paracrine effects of placental mesenchymal cells in an LPS-induced mouse model of PE. LPS was chosen to mimic PE symptoms since it induces generalized endothelial dysfunction via the activation of inflammatory pathways [40]. In accordance with previous reports, in our model, LPS exposure led to gestational hypertension and proteinuria [24,41,42], as observed in PE pregnancies. Importantly, we demonstrated that systemic administration of PDMSC-CM, and not of living cells, significantly reduced LPS-induced gestational hypertension and proteinuria.

In line with our data, other groups reported the efficacy of MSCs in reducing hypertension in vivo [22,24,43]. It was suggested that the mechanism by which MSCs may ameliorate hypertension is through the modulation of endothelium-derived factors that control vasodilatation, vasoconstriction, and microvascular density increase [44]. In two-kidney, one-clip rats, MSCs minimized hypertension at least in part by interfering with sympathetic nerve activity leading to reduction of sympathetic activity in the cardiovascular system [45].

As mentioned above, our results were obtained by injecting PDMSCs conditioned media and not living cells, thus opening the door to less invasive, more ethical, and safe therapeutic approaches for preeclampsia. PDMSCs-CM composition is complex and it includes free proteins, small molecules, and extracellular vesicles which can be further divided into apoptotic bodies, microparticles, and exosomes [46,47]. We previously published PDMSCs-CM partial characterization performed by cytokine array technology [19], demonstrating the presence of cytokines and chemokines in the conditioned media. In particular, we described Interleukin 8 (IL-8), Osteopontin, Tissue Inhibitor of Metalloproteinases-2 (TIMP-2), Neutrophil Activating Peptide 2 (NAP-2), Monocyte Chemotactic Protein-1 (MCP-1), Osteoprotegerin, Transforming Growth Factor-b2 (TGF-b2), Interferon-inducible protein-10 (IP-10), GRO, Vascular Endothelial Growth Factor (VEGF), Placental Growth Factor (PlGF) and Interleukin 10 (IL-10) as components of physiological PDMSCs-CM [19]. Despite that some of these molecules were classified as proinflammatory and Th1 mediators increased in PDMSCs and maternal blood from PE patients, they are also pivotal for physiological embryo implantation, placentation, and maternal–placental vascular remodeling [48,49,50]. For example, IL-8 is able to specifically counteract inflammation at the endothelial level [51], while TIMP-2 and IL-10 are powerful anti-inflammatory molecules, suggesting a multifactorial action directed against the exacerbated inflammation typical of PE. Moreover, since VEGF/sFlt-1 unbalance is widely accepted as the main trigger for the endothelial dysfunction, leading to hypertension in preeclampsia [52,53,54], we hypothesize that PDMSCs-CM counteracted endothelial dysfunction in our LPS-induced PE model via pro-angiogenic VEGF modulation and anti-angiogenic sFlt-1 inhibition. This hypothesis is in line with our previous findings showing that PDMSCs-CM promoted placental VEGF accumulation and sFlt-1 downregulation in physiological human villous explants [19].

Next, we described that PDMSCs-CM administration induced a significant reduction in LPS-induced urinary protein excretion. Similar effects were observed when human placental expanded (PLX-PAD) mesenchymal-like cells were injected in two models of innate immunity-induced PE, thus confirming PDMSCs potential to reduce PE-associated proteinuria [22]. Chatterjee et al. suggested that the release of paracrine factors led to the observed decrease in oxidative stress, angiogenesis, inflammation, and endothelial dysfunction, without the need of cell–cell contact. Since endothelial damage underlies many PE manifestations, including proteinuria [55], our data are in line with the above mentioned results, confirming the ability of human PDMSCs to counteract vascular dysfunction throughout the release of trophic mediators, as VEGF that it is able to stimulate endothelial repair and reduce circulating sFlt-1 levels. In our model, LPS-induced proteinuria slowly decreased with advancing gestation also in animals infused with plain media, even if to a significantly lesser extent relative to CM animals. This effect is likely due to spontaneous LPS clearance and consequent inflammation remission.

Moreover, we reported that LPS followed by plain media infusion in pregnant mice induced reduction in fetuses’ number, fetal absorption, and decrease in fetal and placental weight, thus mimicking placenta development anomalies typical of PE. Similar results were reported by Rivera et al., showing that a 100 μg/kg day LPS for 7 days in pregnant rats significantly reduced fetal size and increased fetal demise [56]. In stark contrast, we demonstrated that PDMSCs-CM treatment resulted in a higher number of fetuses, increased fetal–placental weight, and no fetal absorption. These outcomes are likely due to decreased endothelial damage and placental inflammation as demonstrated by placental sFlt-1, TNF-a, and IL-6 downregulation, reduced maternal blood pressure and proteinuria derived from improved placental functionality, and fetal nutritional status as previously suggested [43,57]. Importantly, we specifically investigated TNF-α, Il-6, and sFlt-1 because they are key players in PE pathogenesis [58] and severe endothelial dysfunction [54,59].

To investigate the impact of PDMSCs-CM on liver and renal functions, serum AST-ALT and creatinine–urea levels were monitored. It was previously described that after MSCs injection (e.g., bone marrow MSC, hematopoietic stem cells, umbilical cords MSC, amniotic fluid MSCs), AST, ALT, creatinine, and urea were normalized to physiological levels restoring compromised liver and kidneys activities [60,61,62]. In our model, AST, ALT, creatinine, and urea serum levels were not modified by LPS injection nor by PDMSCs-CM/plain media infusion, in line with data reported by Oludare and colleagues that described no significant differences in liver enzymes’ levels in LPS mice compared to controls [63]. Importantly, our results demonstrated that PDMSCs-CM did not affect liver and kidney functionalities, thus excluding adverse effects.

In conclusion, our findings demonstrated that human PDMSCs-CM administration significantly ameliorated PE-like symptoms and improved fetal–placental outcomes in pregnant LPS mice. Our data strongly suggested that PDMSCs-CM acted through the restoration of endothelial function and the suppression of the proinflammatory cascade, thus contrasting systemic and placental injury. A limitation of our study was that we have not yet completed PDMSCs conditioned media characterization; therefore, we could not indicate its exact mechanism of action. Due to CM complexity, different pathways were most likely promoted and/or suppressed by PDMSCs trophic mediators, thus explaining the multitarget therapeutic activity demonstrated by our results. Indeed, PDMSCs-CM-based therapy could be considered a promising tool due to its ability to simultaneously target different drivers of the preeclamptic syndrome. This approach could minimize the biological variability, biosafety, and ethical issues of cell-based therapies, thus leading to the development of a safe and ethical cell-free strategy against preeclampsia. Further investigations are required.

## 4. Materials and Methods

### 4.1. PDMSCs Conditioned Media Preparation

PDMSCs-CM was prepared as previously described [19,31]. Briefly, placentae from healthy women with a singleton physiological pregnancy were collected immediately after delivery. Physiological pregnancy was defined as term normotensive pregnancy and no signs of preeclampsia or FGR. Exclusion criteria were congenital malformations, chromosomal abnormalities (in number and/or structure), maternal and/or intrauterine infections, cardiovascular diseases, metabolic syndrome, diabetes, and immunological disorders.

PDMSCs were isolated by enzymatic digestion and gradient as previously described [19,28,31]. PDMSCs were next resuspended in Dulbecco’s Modified Minimum Essential Medium (DMEM, Gibco, Life Technologies, Monza, Italy) supplemented with 10% fetal bovine serum (FBS Australian origin, Life Technologies, Monza, Italy) and maintained at 37 °C and 5% CO_2_. At every passage, physiological PDMSCs were characterized by flow cytometry for the expression of the following antigens: HLA-I, HLA-DR, CD105, C166, CD90, CD34, CD73, CD133, CD20, CD326, CD31, CD45, and CD14 (Miltenyi Biotech, Bologna, Italy). PDMSCs were analyzed by semiquantitative PCR to assess gene expression levels of stem cell markers Oct-4 and Nanog. Primers were designed as previously described [19].

At passage three of culture, after obtaining a pure PDMSCs population, cells were plated and expanded in 1264 cm^2^ EasyFill cell factories (Carlo Erba, Cornaredo (MI), Italy) at a concentration of 3 × 106 cells. When cells reached confluency, media was removed and replaced by 400 mL of DMEM LG without FBS. After 48 h of culture, CM was collected, filtered, and stored at −20 °C until use.

### 4.2. Preeclamptic Mouse Model Preparation and PDMSCs-CM Treatment

The preeclamptic mouse model was prepared following a modified protocol from Wang et al. [57]. Briefly, C57BL/6NCrl virgin mice females (n = 30) and males (n = 10) at 4 weeks of age were purchased from Charles River Laboratories (Calco (LC), Italy). All mice were maintained on a 12 h/12 h dark and light cycle with relative humidity of 50–70% at 18–22 °C. Tap water and standard laboratory pelleted formula were provided.

Female mice were mated with males at 9 weeks of age and plug discovery was considered as day 0 of pregnancy. Female mice lacking copulation plugs (n = 20) were returned to the breeding colony. At day 11 of pregnancy, all pregnant females (n = 10) were removed from breeding cages and received intravenous tail injection of 1 μg/kg LPS solution in order to induce inflammation-mediated endothelial damage and hypertension. At day 12, after blood pressure measurements, mice were randomized into two groups as follows: (1) animals that received a single intravenous tail injection of 300 μL of plain unconditioned media (control group, n = 5); (2) animals that received a single intravenous tail injection of 300 μL PDMSCs-CM (CM group, n = 5) (Figure 1A). LPS was purchased from Sigma Aldrich (Milan, Italy). PDMSCs-CM was prepared as described above. 

Maternal systolic blood pressure (SBP) was monitored by tail cuff plethysmography by using BP-2000 Series II Blood Pressure Analysis System, 2 channels mouse platform (Visitech Systems, Napa Pl, Apex, United States) from day 9 to 18 of pregnancy. Urine samples were collected at days 12, 13, and 17 of pregnancy and analyzed for protein content by Bradford assay (Sigma Aldrich, Milan, Italy). Mice were sacrificed by cervical dislocation at day 19 of pregnancy and uteri were removed. Maternal blood samples were taken from mice carotid and collected in heparin tubes to determine the following hematological parameters: RBC count, WBC count, Plt count, Htc, and Hb. All analyses were performed using a veterinary hematology analyzer. Serum was separated and used for measuring ALT, AST, urea, and creatinine. Placental weights, number of fetuses, and fetal weights were recorded. Placentae were collected and stored at −80 °C until the next molecular analysis. 

### 4.3. RNA Isolation and Real-Time PCR

Total RNA was isolated from PDMSCs-CM-treated and untreated PE placentae using TRIzol reagent (Life Technologies, Invitrogen, Monza, Italy) according to manufacturer instructions. Genomic DNA contamination was removed by DNAse I digestion before RT-PCR. CDNA was generated from 5 μg of total RNA using a random hexamers approach and RevertAid H Minus First Strand cDNA Synthesis kit (Life Technologies, Monza, Italy).

Gene expressions levels of sFlt-1, TNF-α, and IL-6 were determined by real-time PCR using specific TaqMan primers and probes (Life Technologies, Monza, Italy). MRNA levels were normalized using endogenous 18 s as internal reference (Life Technologies, Monza, Italy). Relative expression and fold change were calculated according to Livak and Schmittgen [64].

### 4.4. Enzyme-Linked Immunosorbent Assay (ELISA)

Total proteins were isolated from PDMSCs-CM-treated and untreated PE placentae using 1X radio immunoprecipitation assay (RIPA) buffer. Quantitative measurement of sFlt-1 (R&D System, Milan, Italy), TNF-α (RayBiotech, Prodotti Gianni, Milan, Italy), and IL-6 (Abcam, Milan, Italy) placental levels were determined using commercially available competitive ELISA kits according to manufacturer’s instruction. 

### 4.5. Statistical Analysis

All data are represented as mean ± standard error (SE). For comparison of data between multiple groups, one-way analysis of variance (ANOVA) with post hoc Dunnett’s test was used. For comparison between two groups, Mann–Whitney U-test was used as appropriate. Fisher’s exact test was used for small sample sizes. Statistical analysis was carried out using SPSS Version 23 statistical software, and significance was accepted at *p* < 0.05.

## Figures and Tables

**Table 1:** Effect of PDMSCs-CM treatment on clinical and biochemical parameters of mice in PDMSCs-CM and control groups.

	PDMSCs-CM (n = 5)	Control (n = 5)	*p*-Value
Number of fetuses	41	24	*p* < 0.01
Fetal reabsorption	0	5	*p* = 0.02
Fetal weight, grams (median and range)	0.82 (0.62–1.26)	0.75 (0.59–0.99)	ns
Placental weight, grams (median and range)	0.12 (0.07–0.25)	0.09 (0.05–0.14)	*p* < 0.01
Hematocrit (%)	11.1	10.8	ns
RBC	7.2	7.08	ns
WBC	1.1	1.2	ns
Plt	330	135	ns
Htc (%)	10.4	10.3	ns
Hb	10.1	9.9	ns
ALT (mg/dL)	39.7	39.7	ns
AST (mg/dL)	247.7	263.7	ns
Urea (mg/dL)	35.2	34.2	ns
Creatinine (mg/dL)	0.07	0.07	ns

Significant main effect of PDMSCs-CM treatment on number of fetus, fetal reabsorption, and placental weight. Data are expressed as means ± SEM. ns: not significant.

## Data Availability

The raw data supporting the conclusions of this article will be made available by the authors, without undue reservation.

## References

[1] Ishihara N., Matsuo H., Murakoshi H., Laoag-Fernandez J.B., Samoto T., Maruo T. (2002). Increased Apoptosis in the Syncytiotrophoblast in Human Term Placentas Complicated by Either Preeclampsia or Intrauterine Growth Retardation. Am. J. Obstet. Gynecol..

[2] Redman C.W., Sargent I.L. (2005). Latest Advances in Understanding Preeclampsia. Science.

[3] Cunningham F.G., Lindheimer M.D. (1992). Hypertension in Pregnancy. N. Engl. J. Med..

[4] Pennington K.A., Schlitt J.M., Jackson D.L., Schulz L.C., Schust D.J. (2012). Preeclampsia: Multiple Approaches for a Multifactorial Disease. Dis. Model. Mech..

[5] Marzi M., Vigano A., Trabattoni D., Villa M.L., Salvaggio A., Clerici E., Clerici M. (1996). Characterization of Type 1 and Type 2 Cytokine Production Profile in Physiologic and Pathologic Human Pregnancy. Clin. Exp. Immunol..

[6] Saito S., Sakai M., Sasaki Y., Tanebe K., Tsuda H., Michimata T. (1999). Quantitative Analysis of Peripheral Blood Th0, Th1, Th2 and the Th1:Th2 Cell Ratio during Normal Human Pregnancy and Preeclampsia. Clin. Exp. Immunol..

[7] Saito S., Umekage H., Sakamoto Y., Sakai M., Tanebe K., Sasaki Y., Morikawa H. (1999). Increased T-Helper-1-Type Immunity and Decreased T-Helper-2-Type Immunity in Patients with Preeclampsia. Am. J. Reprod. Immunol..

[8] Pijnenborg R., Vercruysse L., Verbist L., Van Assche F.A. (1998). Interaction of Interstitial Trophoblast with Placental Bed Capillaries and Venules of Normotensive and Pre-Eclamptic Pregnancies. Placenta.

[9] Sibai B., Dekker G., Kupferminc M. (2005). Pre-Eclampsia. Lancet.

[10] Gammill H.S., Roberts J.M. (2007). Emerging Concepts in Preeclampsia Investigation. Front. Biosci..

[11] Lockwood C.J., Yen C.-F., Basar M., Kayisli U.A., Martel M., Buhimschi I., Buhimschi C., Huang S.J., Krikun G., Schatz F. (2008). Preeclampsia-Related Inflammatory Cytokines Regulate Interleukin-6 Expression in Human Decidual Cells. Am. J. Pathol..

[12] Mandò C., Razini P., Novielli C., Anelli G.M., Belicchi M., Erratico S., Banfi S., Meregalli M., Tavelli A., Baccarin M. (2016). Impaired Angiogenic Potential of Human Placental Mesenchymal Stromal Cells in Intrauterine Growth Restriction. Stem Cells Transl. Med..

[13] Yen B.L., Huang H.-I., Chien C.-C., Jui H.-Y., Ko B.-S., Yao M., Shun C.-T., Yen M.-L., Lee M.-C., Chen Y.-C. (2005). Isolation of Multipotent Cells from Human Term Placenta. Stem Cells.

[14] Brooke G., Tong H., Levesque J.-P., Atkinson K. (2008). Molecular Trafficking Mechanisms of Multipotent Mesenchymal Stem Cells Derived from Human Bone Marrow and Placenta. Stem Cells Dev..

[15] Parolini O., Alviano F., Bergwerf I., Boraschi D., De Bari C., De Waele P., Dominici M., Evangelista M., Falk W., Hennerbichler S. (2010). Toward Cell Therapy Using Placenta-Derived Cells: Disease Mechanisms, Cell Biology, Preclinical Studies, and Regulatory Aspects at the Round Table. Stem Cells Dev..

[16] Fukuchi Y., Nakajima H., Sugiyama D., Hirose I., Kitamura T., Tsuji K. (2004). Human Placenta-Derived Cells Have Mesenchymal Stem/Progenitor Cell Potential. Stem Cells.

[17] Chang C.-J., Yen M.-L., Chen Y.-C., Chien C.-C., Huang H.-I., Bai C.-H., Yen B.L. (2006). Placenta-Derived Multipotent Cells Exhibit Immunosuppressive Properties That Are Enhanced in the Presence of Interferon-Gamma. Stem Cells.

[18] Li C., Zhang W., Jiang X., Mao N. (2007). Human-Placenta-Derived Mesenchymal Stem Cells Inhibit Proliferation and Function of Allogeneic Immune Cells. Cell Tissue Res..

[19] Rolfo A., Giuffrida D., Nuzzo A.M., Pierobon D., Cardaropoli S., Piccoli E., Giovarelli M., Todros T. (2013). Pro-Inflammatory Profile of Preeclamptic Placental Mesenchymal Stromal Cells: New Insights into the Etiopathogenesis of Preeclampsia. PLoS ONE.

[20] Liu L., Zhao G., Fan H., Zhao X., Li P., Wang Z., Hu Y., Hou Y. (2014). Mesenchymal Stem Cells Ameliorate Th1-Induced Pre-Eclampsia-like Symptoms in Mice via the Suppression of TNF-α Expression. PLoS ONE.

[21] Fu L., Liu Y., Zhang D., Xie J., Guan H., Shang T. (2015). Beneficial Effect of Human Umbilical Cord-Derived Mesenchymal Stem Cells on an Endotoxin-Induced Rat Model of Preeclampsia. Exp. Ther. Med..

[22] Chatterjee P., Chiasson V.L., Pinzur L., Raveh S., Abraham E., Jones K.A., Bounds K.R., Ofir R., Flaishon L., Chajut A. (2016). Human Placenta-Derived Stromal Cells Decrease Inflammation, Placental Injury and Blood Pressure in Hypertensive Pregnant Mice. Clin. Sci..

[23] Dominina A.P., Fridliandskaia I.I., Zemel’ko V.I., Pugovkina N.A., Kovaleva Z.V., Zenin V.V., Grinchuk T.M., Nikol’skiĭ N.N. (2013). Mesenchymal stem cells of human endometrium do not undergo spontaneous transformation during long-term cultivation. Tsitologiia.

[24] Wang S., Qu X., Zhao R.C. (2012). Clinical Applications of Mesenchymal Stem Cells. J. Hematol. Oncol..

[25] Wu W., He Q., Li X., Zhang X., Lu A., Ge R., Zhen H., Chang A.E., Li Q., Shen L. (2011). Long-Term Cultured Human Neural Stem Cells Undergo Spontaneous Transformation to Tumor-Initiating Cells. Int. J. Biol. Sci..

[26] Bernardo M.E., Zaffaroni N., Novara F., Cometa A.M., Avanzini M.A., Moretta A., Montagna D., Maccario R., Villa R., Daidone M.G. (2007). Human Bone Marrow Derived Mesenchymal Stem Cells Do Not Undergo Transformation after Long-Term in Vitro Culture and Do Not Exhibit Telomere Maintenance Mechanisms. Cancer Res..

[27] Popov B.V., Petrov N.S., Mikhailov V.M., Tomilin A.N., Alekseenko L.L., Grinchuk T.M., Zaichik A.M. (2009). Spontaneous Transformation and Immortalization of Mesenchymal Stem Cells in Vitro. Cell Tissue Biol..

[28] Nuzzo A.M., Giuffrida D., Zenerino C., Piazzese A., Olearo E., Todros T., Rolfo A. (2014). JunB/Cyclin-D1 Imbalance in Placental Mesenchymal Stromal Cells Derived from Preeclamptic Pregnancies with Fetal-Placental Compromise. Placenta.

[29] Karp J.M., Leng Teo G.S. (2009). Mesenchymal Stem Cell Homing: The Devil Is in the Details. Cell Stem Cell.

[30] Asami T., Ishii M., Fujii H., Namkoong H., Tasaka S., Matsushita K., Ishii K., Yagi K., Fujiwara H., Funatsu Y. (2013). Modulation of Murine Macrophage TLR7/8-Mediated Cytokine Expression by Mesenchymal Stem Cell-Conditioned Medium. Mediat. Inflamm..

[31] Nuzzo A.M., Giuffrida D., Masturzo B., Mele P., Piccoli E., Eva C., Todros T., Rolfo A. (2017). Altered Expression of G1/S Phase Cell Cycle Regulators in Placental Mesenchymal Stromal Cells Derived from Preeclamptic Pregnancies with Fetal-Placental Compromise. Cell Cycle.

[32] Peng C.-K., Wu S.-Y., Tang S.-E., Li M.-H., Lin S.-S., Chu S.-J., Huang K.-L. (2017). Protective Effects of Neural Crest-Derived Stem Cell-Conditioned Media against Ischemia-Reperfusion-Induced Lung Injury in Rats. Inflammation.

[33] Yang C., Lei D., Ouyang W., Ren J., Li H., Hu J., Huang S. (2014). Conditioned Media from Human Adipose Tissue-Derived Mesenchymal Stem Cells and Umbilical Cord-Derived Mesenchymal Stem Cells Efficiently Induced the Apoptosis and Differentiation in Human Glioma Cell Lines in Vitro. BioMed. Res. Int..

[34] Zuk P.A., Zhu M., Ashjian P., De Ugarte D.A., Huang J.I., Mizuno H., Alfonso Z.C., Fraser J.K., Benhaim P., Hedrick M.H. (2002). Human Adipose Tissue Is a Source of Multipotent Stem Cells. Mol. Biol. Cell.

[35] Lee O.K., Kuo T.K., Chen W.-M., Lee K.-D., Hsieh S.-L., Chen T.-H. (2004). Isolation of Multipotent Mesenchymal Stem Cells from Umbilical Cord Blood. Blood.

[36] Hu Y., Liao L., Wang Q., Ma L., Ma G., Jiang X., Zhao R.C. (2003). Isolation and Identification of Mesenchymal Stem Cells from Human Fetal Pancreas. J. Lab. Clin. Med..

[37] Abomaray F.M., Al Jumah M.A., Kalionis B., AlAskar A.S., Al Harthy S., Jawdat D., Al Khaldi A., Alkushi A., Knawy B.A., Abumaree M.H. (2015). Human Chorionic Villous Mesenchymal Stem Cells Modify the Functions of Human Dendritic Cells, and Induce an Anti-Inflammatory Phenotype in CD1+ Dendritic Cells. Stem Cell Rev. Rep..

[38] Abumaree M.H., Abomaray F.M., Alshabibi M.A., AlAskar A.S., Kalionis B. (2017). Immunomodulatory Properties of Human Placental Mesenchymal Stem/Stromal Cells. Placenta.

[39] Du W., Li X., Chi Y., Ma F., Li Z., Yang S., Song B., Cui J., Ma T., Li J. (2016). VCAM-1+ Placenta Chorionic Villi-Derived Mesenchymal Stem Cells Display Potent pro-Angiogenic Activity. Stem Cell Res. Ther..

[40] Grylls A., Seidler K., Neil J. (2021). Link between Microbiota and Hypertension: Focus on LPS/TLR4 Pathway in Endothelial Dysfunction and Vascular Inflammation, and Therapeutic Implication of Probiotics. Biomed. Pharmacother..

[41] Ding X., Yang Z., Han Y., Yu H. (2014). Fatty Acid Oxidation Changes and the Correlation with Oxidative Stress in Different Preeclampsia-like Mouse Models. PLoS ONE.

[42] Lin F., Zeng P., Xu Z., Ye D., Yu X., Wang N., Tang J., Zhou Y., Huang Y. (2012). Treatment of Lipoxin A(4) and Its Analogue on Low-Dose Endotoxin Induced Preeclampsia in Rat and Possible Mechanisms. Reprod. Toxicol..

[43] Zhang D., Fu L., Wang L., Lin L., Yu L., Zhang L., Shang T. (2017). Therapeutic Benefit of Mesenchymal Stem Cells in Pregnant Rats with Angiotensin Receptor Agonistic Autoantibody-Induced Hypertension: Implications for Immunomodulation and Cytoprotection. Hypertens. Pregnancy.

[44] De Oliveira L.F., Almeida T.R., Ribeiro Machado M.P., Cuba M.B., Alves A.C., da Silva M.V., Rodrigues Júnior V., Dias da Silva V.J. (2015). Priming Mesenchymal Stem Cells with Endothelial Growth Medium Boosts Stem Cell Therapy for Systemic Arterial Hypertension. Stem Cells Int..

[45] Oliveira-Sales E.B., Maquigussa E., Semedo P., Pereira L.G., Ferreira V.M., Câmara N.O., Bergamaschi C.T., Campos R.R., Boim M.A. (2013). Mesenchymal Stem Cells (MSC) Prevented the Progression of Renovascular Hypertension, Improved Renal Function and Architecture. PLoS ONE.

[46] Vizoso F.J., Eiro N., Cid S., Schneider J., Perez-Fernandez R. (2017). Mesenchymal Stem Cell Secretome: Toward Cell-Free Therapeutic Strategies in Regenerative Medicine. Int. J. Mol. Sci..

[47] Beer L., Mildner M., Ankersmit H.J. (2017). Cell Secretome Based Drug Substances in Regenerative Medicine: When Regulatory Affairs Meet Basic Science. Ann. Transl. Med..

[48] Albonici L., Benvenuto M., Focaccetti C., Cifaldi L., Miele M.T., Limana F., Manzari V., Bei R. (2020). PlGF Immunological Impact during Pregnancy. Int. J. Mol. Sci..

[49] Jones R.L., Stoikos C., Findlay J.K., Salamonsen L.A. (2006). TGF-Beta Superfamily Expression and Actions in the Endometrium and Placenta. Reprod. Camb. Engl..

[50] Wu L.-Z., Liu X.-L., Xie Q.-Z. (2015). Osteopontin Facilitates Invasion in Human Trophoblastic Cells via Promoting Matrix Metalloproteinase-9 in Vitro. Int. J. Clin. Exp. Pathol..

[51] Qazi B.S., Tang K., Qazi A. (2011). Recent advances in underlying pathologies provide insight into interleukin-8 expression-mediated inflammation and angiogenesis. Int. J. Inflam..

[52] Tang Y., Ye W., Liu X., Lv Y., Yao C., Wei J. (2019). VEGF and SFLT-1 in Serum of PIH Patients and Effects on the Foetus. Exp. Ther. Med..

[53] Nuzzo A.M., Giuffrida D., Moretti L., Re P., Grassi G., Menato G., Rolfo A. (2021). Placental and Maternal SFlt1/PlGF Expression in Gestational Diabetes Mellitus. Sci. Rep..

[54] Rolfo A., Attini R., Nuzzo A.M., Piazzese A., Parisi S., Ferraresi M., Todros T., Piccoli G.B. (2013). Chronic Kidney Disease May Be Differentially Diagnosed from Preeclampsia by Serum Biomarkers. Kidney Int..

[55] Ali S.M.J., Khalil R.A. (2015). Genetic, Immune and Vasoactive Factors in the Vascular Dysfunction Associated with Hypertension in Pregnancy. Expert Opin. Ther. Targets.

[56] Rivera D.L., Olister S.M., Liu X., Thompson J.H., Zhang X.J., Pennline K., Azuero R., Clark D.A., Miller M.J. (1998). Interleukin-10 Attenuates Experimental Fetal Growth Restriction and Demise. FASEB J. Off. Publ. Fed. Am. Soc. Exp. Biol..

[57] Vonlaufen A., Phillips P.A., Xu Z., Zhang X., Yang L., Pirola R.C., Wilson J.S., Apte M.V. (2011). Withdrawal of Alcohol Promotes Regression While Continued Alcohol Intake Promotes Persistence of LPS-Induced Pancreatic Injury in Alcohol-Fed Rats. Gut.

[58] Wang L.-L., Yu Y., Guan H.-B., Qiao C. (2016). Effect of Human Umbilical Cord Mesenchymal Stem Cell Transplantation in a Rat Model of Preeclampsia. Reprod. Sci..

[59] Zhang Z., Dai M. (2014). Effect of paeonol on adhesive function of rat vascular endothelial cells induced by lipopolysaccharide and co-cultured with smooth muscle cells. Zhongguo Zhong Yao Za Zhi Zhongguo Zhongyao Zazhi/China J. Chin. Mater. Med..

[60] Zekri A.-R.N., Salama H., Medhat E., Musa S., Abdel-Haleem H., Ahmed O.S., Khedr H.A.H., Lotfy M.M., Zachariah K.S., Bahnassy A.A. (2015). The Impact of Repeated Autologous Infusion of Haematopoietic Stem Cells in Patients with Liver Insufficiency. Stem Cell Res. Ther..

[61] Shi M., Liu Z., Wang Y., Xu R., Sun Y., Zhang M., Yu X., Wang H., Meng L., Su H. (2017). A Pilot Study of Mesenchymal Stem Cell Therapy for Acute Liver Allograft Rejection. Stem Cells Transl. Med..

[62] Hauser P.V., De Fazio R., Bruno S., Sdei S., Grange C., Bussolati B., Benedetto C., Camussi G. (2010). Stem Cells Derived from Human Amniotic Fluid Contribute to Acute Kidney Injury Recovery. Am. J. Pathol..

[63] Oludare G.O., Ilo O.J., Lamidi B.A. (2017). Effects of Lipopolysaccharide and High Saline Intake on Blood Pressure, Angiogenic Factors and Liver Enzymes of Pregnant Rats. Niger. J. Physiol. Sci. Off. Publ. Physiol. Soc. Niger..

[64] Livak K.J., Schmittgen T.D. (2001). Analysis of Relative Gene Expression Data Using Real-Time Quantitative PCR and the 2(-Delta Delta C(T)) Method. Methods San Diego Calif.

